# Fractalkine Is Linked to the Necrosome Pathway in Acute Pulmonary Inflammation

**DOI:** 10.3389/fmed.2021.591790

**Published:** 2021-03-12

**Authors:** Kristian-Christos Ngamsri, Jutta Gamper-Tsigaras, Jörg Reutershan, Franziska M. Konrad

**Affiliations:** ^1^Department of Anesthesiology and Intensive Care Medicine, University Hospital of Tübingen, Tübingen, Germany; ^2^Department of Anesthesiology and Intensive Care Medicine, Hospital of Bayreuth, Bayreuth, Germany

**Keywords:** migration, acute inflammation, necroptosis, neutrophil (PMN), CX3CL1 (fractalkine), CX3CR1

## Abstract

Acute pulmonary inflammation affects over 10% of intensive care unit (ICU) patients and is associated with high mortality. Fractalkine (CX_3_CL1) and its receptor, CX_3_CR1, have been shown to affect pulmonary inflammation, but previous studies have focused on macrophages. In a murine model of acute pulmonary inflammation, we identified inflammatory hallmarks in C57BL/6J and CX_3_CR1^−/−^ mice. Pulmonary inflammation was significantly enhanced in the CX_3_CR1^−/−^ animals compared to the C57BL/6J animals, as assessed by microvascular permeability, polymorphonuclear neutrophil (PMN) migration into lung tissue and alveolar space. The CX_3_CR1^−/−^ mice showed increased levels of apoptotic PMNs in the lungs, and further investigations revealed an increased activation of necrosome-related receptor-interacting serine/threonine-protein kinases 1 (RIPK1), 3 (RIPK3), and mixed-lineage kinase domain-like pseudokinase (MLKL). Phosphorylated MLKL leads to membrane rupture and damage-associated molecular pattern (DAMP) release, which further enhance inflammation. The release of DAMPs was significantly higher in the CX_3_CR1^−/−^ mice and led to the activation of various cascades, explaining the increased inflammation. RIPK3 and MLKL inhibition improved the inflammatory response in human PMNs *in vitro* and confirmed our *in vivo* findings. In conclusion, we linked CX_3_CL1 to the necrosome complex in pulmonary inflammation and demonstrated a pivotal role of the necrosome complex in human PMNs.

## Introduction

Inflammation is a tightly regulated process that is characterized by the synchronized activation and orchestration of immune cells. Inflammation is caused by either tissue injury or infection, and its main function is eliminating the pathogenic insult or the damaged tissue. Both injury and infection induce the release of chemotactic factors to recruit polymorphonuclear neutrophils (PMNs) to the site of inflammation (1). Therefore, PMNs are the first cells to be recruited to the site of infection. In the case of pulmonary inflammation, PMNs migrate from the circulation into the lung interstitium, passing through an endothelial barrier followed by an epithelial barrier and migrating into the alveolar space. Although PMNs are necessary for defense, their excessive migration into inflamed tissue even exacerbates tissue damage (2), particularly through their release of reactive oxygen species (ROS) as part of their defense mechanisms (3). The inefficient clearance of affected cells maintains inflammation and disrupts tissue homeostasis (4).

Recent studies have suggested that apoptosis and necroptosis play a critical role in pulmonary inflammation (5–7). Both apoptosis and necroptosis are distinct cell death pathways that are differentially regulated. In contrast to apoptotic cell death, necrotic cell death that is caused by infection or tissue injury leads to the disruption of cell membranes and the release of cytoplasmic and nuclear components that act as damage-associated molecular patterns (DAMPs) (8), further exacerbating inflammation; therefore, necrosis is considered pro-inflammatory (9). However, it is methodically difficult to determine if PMNs are undergoing late apoptosis or necroptosis (10). During necroptosis, Toll-like receptor 4 (TLR4) activation leads to the phosphorylation of homologous serine/threonine kinase (RIPK) 1, which recruits and activates RIPK3. Together, RIPK1 and RIPK3 form an insoluble aggregate termed the “necrosome,” which causes the phosphorylation of mixed-lineage kinase domain-like pseudokinase (MLKL). The phosphorylation, and therefore activation, of MLKL promotes its translocation to the cell surface, leading to membrane rupture and DAMP release to the extracellular space, where innate immune cells, such as macrophages and dendritic cells, are located. Innate immune cells recognize DAMPs during infection or tissue damage to evoke an inflammatory cytokine response. Some DAMPs that are released by the necrosome are high motility group box 1 (HMGB1) and alarmins, which are molecules that perform cytokine-like functions (e.g., IL-1α and IL-33) (11, 12). Recent investigations suggest a connection between the chemokine receptor CX_3_CR1 and the necrosome complex (13). The chemokine CX_3_CL1, also named fractalkine, is a transmembrane molecule that also exists in soluble form; the soluble form consists of a chemokine domain and performs a chemoattractant function, while the membrane-bound form functions as an adhesion molecule. The only known receptor of CX_3_CL1 is CX_3_CR1, which is expressed on lymphocytes, macrophages, and platelets (14–16) and plays a crucial role in pulmonary inflammation (17). In the current study, we investigated the role of the CX_3_CL1-CX_3_CR1 axis in pulmonary inflammation in detail with respect to PMN migration, chemokine release and microvascular permeability. Furthermore, we analyzed the link between the CX_3_CL1-CX_3_CR1 axis and the necrosome complex.

## Materials and Methods

### Animals

C57BL/6J mice (male; 8–12 weeks old) were obtained from Charles River Laboratories (Sulzberg). B6·129P-CX_3_CR1tm1Litt/J male mice homozygous for the CX_3_CR1-GFP targeted mutation (referred to as CX_3_CR1^−/−^ mice) were purchased from Jackson Laboratories (Bar Harbor) and were bred for the animal experiments. For all the surgical processes, the animals were anesthetized with ketamine and xylazine. All the animal protocols were approved by the Animal Care and Use Committee of the University of Tuebingen.

### Murine Model of Pulmonary Inflammation

As previously described in detail, the mice were exposed to nebulized LPS (*Salmonella enteritidis*; Sigma–Aldrich; 7 ml in total; 500 μg/ml) in a custom-made chamber for 45 min (18). LPS exposure led to reproducible effects of pulmonary inflammation, such as transmigration of leukocytes, changes in microvascular permeability and release of inflammatory chemokines. The control mice inhaled a 0.9% NaCl solution. The specific function-blocking CX_3_CR1 antagonist (5 mg/kg bw; TP501; Torrey Pine Biolabs) and the specific function-blocking CX_3_CL1 antagonist (5 mg/kg bw; TP233; Torrey Pine Biolabs) were administered intraperitoneally 1 h before LPS inhalation. The control mice received intraperitoneally an equivalent dose of IgG1-control antibody (Torrey Pine Biolabs).

### *In vivo* Migration Assay

Three and 24 h after LPS exposure, the mice were anesthetized. The migration of PMNs into the different compartments of the lung (attached to the pulmonary endothelium, interstitial and intra-alveolar) was determined by a flow cytometry-based method. A fluorescent APC-conjugated Ly6G antibody (clone 1A8) was injected into the tail vein to label the intravascular PMNs in the pulmonary vasculature. Under deep anesthesia, a thoracotomy was performed, and 2 ml of saline was injected into the beating heart to remove the blood-free lung and non-adherent leukocytes. Tracheal incision was conducted, and 2 ml PBS was injected to harvest the BAL. The lungs were homogenized and incubated with fluorescent antibodies against anti-CD45 PerCP-Cy5·5 (clone 30-F11) and anti-Ly6G PE/Cy7 (clone 1A8) (19). The absolute cell counts in the BAL and lungs were determined. We differentiated between interstitial PMNs (CD45-PerCP-Cy5·5^+^; Ly6G-PE-Cy7^+^; and Ly6G-APC^−^) and intravascular PMNs (CD45-PerCP-Cy5·5^+^; Ly6G-PE-Cy7^+^; and Ly6G-APC^+^) by flow cytometry (FACS Canto II; BD Biosciences). The gating process is shown in Supplementary Figure 1A. Additionally, we evaluated the surface expression of CCR2 (clone SA203G11) on PMNs (all the antibodies were purchased from BioLegend).

### Neutrophil Isolation From Murine Bone Marrow

Murine bone marrow was flushed from the femurs and tibias of the C57BL/6J and CX_3_CR1^−/−^ animals. Neutrophils were isolated by using histopaque 10831 and 1119 (Sigma-Aldrich). The neutrophils were washed, counted, and purified (>95%), and the viability was determined by flow cytometry (Supplementary Figure 1B).

### Neutrophil MPO and ROS Assay

As a marker of leukocyte activity, we evaluated the concentration of myeloperoxidase (MPO) in the BAL 3 and 24 h after LPS administration. Additionally, MPO release from isolated murine neutrophils from bone marrow and human neutrophils from healthy volunteers was determined. MPO was measured colorimetrically at 405 nm by a plate reader (Tecan Reader Infinite M200PRO). Reactive oxygen species (ROS) were detected by dihydroethidium (D7008; Sigma-Aldrich) with a flow cytometry-based method under the indicated conditions (19).

### Necroptotic/Late Apoptotic PMNs

In additional experiments, we evaluated the apoptosis of PMNs *in vivo* by Annexin V/7-ADD staining. Cell suspensions from BAL and lung tissue were isolated as described above, and the PMN cell surface was labeled by staining with Ly6G-APC. All the cells were pretreated with a specific binding buffer, followed by Annexin V/7-ADD staining. After incubation, the cell suspensions were immediately analyzed by flow cytometry (Supplementary Figure 1C).

Isolated human PMNs (Percoll gradient; GE Healthcare Bio-Sciences; USA) were treated with LPS (100 ng/ml) for 4 and 24 h to induce necroptosis/apoptosis. We labeled the PMN cell surface by CD66b-BV421 (clone G10F5; BD Biosciences) and identified the necroptotic/late apoptotic neutrophils by using Annexin V/7-ADD staining (Supplementary Figure 1D). For the *in vitro* experiments, as described above, stimulated human PMNs were incubated with MM6 cells to induce the clearance of necroptotic/late apoptotic PMNs. Then, we labeled the PMNs with CD11b-BV421 (clone ICRF44)/CD66b-FITC (clone G10F5) and labeled the MM6 cells with CD11b-BV421 (clone ICRF44)/CD14-APC (clone 63D3). Subsequently, we determined the clearance of human PMNs by MM6 cells by permeabilization and staining with CD66b-PE-Cy7 (clone G10F5) by flow cytometry (all the antibodies were purchased from BioLegend). In additional experiments, MM6 cells (MM6; ACC124; DSMZ Braunschweig) were pretreated with human anti-CX_3_CR1 (20 μg/ml; TP502; Torrey Pine Biolabs) or CX_3_CR1 siRNA (sc-39904; Santa Cruz) to evaluate the effects on the clearance of necroptotic/late apoptotic PMNs. After depletion by siRNA, the CX3CL1 gene levels (Supplementary Figure 2A) and CX_3_CR1 surface expression (Supplementary Figure 2B) were determined as controls. Non-targeting siRNA (sc-37007; Santa Cruz) was used as a control. The non-specific phagocytosis capacity was assessed by the quantification of FITC-conjugated dextran clearance (46945; Sigma-Aldrich).

Furthermore, we evaluated the effects of the specific inhibition of RIPK1 (300 nM; GSK'481; 2608; Axon Medchem), RIPK3 (3 μM; GSK'872; 530389; Merck) and MLKL (1 μM; Necrosulfonamide; NSA; S8251; Selleckchem) on murine and human PMNs.

### Determination of Microvascular Permeability

Protein extravasation in the BAL of C57BL/6J and CX_3_CR1 gene-deficient mice after LPS inhalation was evaluated by the BCA protein assay kit according to the standard protocol (Pierce™; Thermo Fisher) (19). Additionally, we determined the Evans blue extravasation in murine lungs colorimetrically. Evans blue (20 mg/kg bw; Sigma-Aldrich) was injected into the tail vein 6 h after LPS or NaCl 0.9% exposure. We performed a median thoracotomy and harvested and homogenized the lungs. Using formamide, we extracted pulmonary Evans blue, and the final concentrations were assessed using a plate reader (20).

### RT-PCR

Total RNA was isolated from the murine lung samples, bone marrow neutrophils, and human PMNs by using pegGOLD TriFast™ (Peglab). cDNA synthesis was performed according to the manufacturer's direction (Bio-Rad iSkript-kit). The gene, gene bank number and primer sequences are given in Tables 1A,B. Data analysis was performed using the 2-ΔCT method (CFX-Manager; BioRad) for relative expression, and all the values were normalized to the 18S expression values as the housekeeping gene.

**Table 1:** List of murine and human primer sequences used for RT-PCR analysis.

**Gene**	**Gene bank number**	**Primer sequence (5^**′**^-3^**′**^)**
**(A)**		
CX_3_CL1	NM_009142.3	F: ACGAAATGCGAAATCATGTGCR: CTGTGTCGTCTCCAGGACAA
TNFα	NM_013693.3	F: TCTTCTCATTCCTGCTTGTGGR: GATCTGAGTGTGAGGGTCTGG
CXCL1	NM_008176.3	F: AAACCGAAGTCATAGCCACACR: GGGGACACCTTTTAGCATCTT
CXCL2/3	NM_009140.2	F: ATCCAGAGCTTGAGTGTGACGR: GCCTTGCCTTTGTTCAGTATC
CCL2	NM_011333.3	F: CAGGTCCCTGTCATGCTTCTR: GTGGGGCGTTAACTGCATCT
CCL5	NM_0136553.3	F: TGCTCCAATCTTGCAGTCGTR: GCAAGCAATGACAGGGAAGC
CCR2	NM_009915.2	F: GCCATCATAAAGGAGCCATACCR: AGGGAGTAGAGTGGAGGCAG
RIPK1	NM_009068.3	F: CCGAGCAGGTCAAATTCAGR: CACACTGCGATCATTCTCG
RIPK3	NM_019955.2	F: CGCTTTAGAAGCCTTCAGGTTGACR: GCAGGCTCTGGTGACAAGATTCATG
IL33	NM_133775.3	F: GATGGGAAGAAGCTGATGGTGR: TTGTGAAGGACGAAGAAGGC
18s (housekeeping gene)	NC_000021.9	F: GTAACCCGTTGAACCCCATTR: CCATCCAATCGGTAGCG
**(B)**		
RIPK1	NM_001354931.2	F: GCACAGCAAAGACCTTACGR: TGTTCCAAAGCCATGTGAG
RIPK3	NM_006871.4	F: CAAGGAGGGACAGAAATGGR: TTGTGGAACCTGCTCCTCT
IL33	NM_033439.4	F: GAACACAGCAAGCAAAGCR: CCAAAGGCAAAGCACTCC
18s (housekeeping gene)	NC_000021.9	F: GTAACCCGTTGAACCCCATTR: CCATCCAATCGGTAGCG

*List of murine **(A)** and human **(B)** gene names, gene bank numbers, and primer sequences used in this study for RT-PCR experiments (F, forward primer; R, reverse primer)*.

### Western Blot

Murine lungs from C57BL/6J and CX_3_CR1^−/−^ mice were removed and prepared for western blot analysis. The protein concentration was detected according to the standard protocol of the protein assay kit (Pierce™; Thermo Fisher). After equalizing the protein levels, the samples were loaded on SDS gels. After blotting onto PVDF membranes, anti-TRIF (PA5-23467; Thermo Fisher), anti-TRAF2 (sc-877; Santa Cruz) (Supplementary Figure 3A), anti-TRAF6 (sc-7221; Santa Cruz) (Supplementary Figure 3A), anti-RIP1 (PA5-20811; Thermo Fisher), anti-RIP3 (ab56164; Abcam), anti-phospho-RIP1 (Ser166) (#31122; Cell Signaling Technology), anti-phospho-RIP3 (Thr231/Ser232) (#57220; Cell Signaling Technology), anti-phospho-MLKL (ab196436; Abcam), anti-AKT1/2 (sc-1619; Santa Cruz) (Supplementary Figure 3A), anti-p-AKT1/2/3 (sc-7985; Santa Cruz), ERK (sc-93; Santa Cruz) (Supplementary Figure 3A), anti-pERK1/2 (sc-7383; Santa Cruz), anti-NFκB p65 (sc-372; Santa Cruz), anti-zonula occludens (ZO)-1 (61-7300; Thermo Fisher), anti-occludin (33-1500; Thermo Fisher), and anti-NFκB p50/p105 (ab32360; Abcam) antibodies were utilized. For the detection of human proteins, we used anti-TRIF (PA5-23467; Thermo Fisher), anti-RIPK1 (PA5-20811; Thermo Fisher), anti-RIPK3 (PA5-28601; Thermo Fisher), anti-phospho-RIP1 (Ser166) (#65746; Cell Signaling Technology), anti-phospho-RIP3 (Ser227) (#93654; Cell Signaling Technology), and anti-phospho-MLKL (#91689; Cell Signaling Technology) antibodies.

Anti-GAPDH served as a housekeeping protein (sc-25778; Santa Cruz). Intensity analysis of the western blots was performed by ImageJ Free-Software (Version 1.51r; NIH).

### Cytokine Release

The release of murine TNFα, CXCL1, CXCL2/3, IL-33 (all R&D Systems), and HMGB1 (E-20749; Qayee-Bio) in the BAL of mice after LPS inhalation was determined by enzyme-linked immunosorbent assay kits according to the manufacturer's instructions.

Additionally, we detected IL-33 and HMGB1 from the supernatants of bone marrow neutrophils under the indicated conditions. Human IL-33 (R&D Systems) and HMGB1 (LS-F4038-1; LS-Bio) were measured from the supernatants of human PMNs. Western blots intensity was measured by ImageJ (Version 1.49v; National Institute of Health; USA).

### Immunohistochemistry and Immunofluorescence Staining

Immunohistochemistry of PMNs in lung sections was performed using a Vectastain ABC Kit (Linaris; Germany). The sections were blocked with an avidin solution (Vector Labs) for 1 h, followed by incubation with an anti-mouse Ly6G antibody (ab25377; clone RB6-8C5; Abcam) or IgG control (sc-2026; Santa Cruz) overnight. The sections were incubated with biotinylated anti-rat IgG (BA-4000; Vector Labs) for half an hour followed by Vectastain ABC reagent (PK-4000; Vector Labs). Images were captured with a Leitz DM IRB microscope (Leica) and analyzed with AxioVision v4.8.2. For the evaluation of septal wall thickness, the lung tissue sections were stained with hematoxylin and eosin. Random sections from four different lungs in each group were examined in a blinded manner by an independent researcher with AxioVision and evaluated with ImageJ. We performed negative controls, respectively, IgG and secondary antibody staining experiments as controls (Supplementary Figure 4B).

For immunofluorescence staining, the paraffin-embedded lung sections were fixed for 10 min in acetone and methanol. After washing, the lung sections were permeabilized with 1% Triton X and blocked with 5% BSA in PBS- for 1 h. The sections were stained with rabbit anti-CX_3_CL1 (TP233; Torrey Pines Biolabs), rabbit anti-CCR2 antibody (ab273050) (Supplementary Figure 4C), anti-mouse Ly6G (ab25377; clone RB6-8C5; Abcam) and anti-mouse phospho-MLKL (ab196436; Abcam) antibodies. Bone marrow neutrophils from the C57BL/6J and CX_3_CR1^−/−^ animals were stimulated with LPS for 4 h (100 ng/ml; *E. coli*; Sigma-Aldrich), fixed, and permeabilized. Subsequently, the cells were blocked with 5% BSA for 1 h and stained with rabbit anti-CX_3_CL1 (TP233; Torrey Pines Biolabs) and anti-mouse Ly6G (ab25377; clone RB6-8C5; Abcam) antibodies. Human PMNs were isolated and stimulated for 4 h with LPS. Subsequently, PMNs were fixed, permeabilized, and blocked. For staining, we used anti-human phospho-RIPK (#44590; Cell Signaling Technology), anti-human phospho-RIPK3 (#93654; Cell Signaling Technology), and anti-human phospho-MLKL (#91689; Cell Signaling Technology) antibodies. The following secondary antibodies were used to label the primary antibodies and to visualize the targets: IgG Alexa 488 (sc-362261; Santa Cruz), IgG Alexa Fluor 546 (A11081; Thermo Fisher), IgG Alexa Fluor 546 (A21085; Thermo Fisher), and IgG Alexa Fluor 546 (A11010; Thermo Fisher). For nuclear counterstaining, Roti-Mount FluorCare DAPI (HP20·1; Carl Roth) was used. For IgG controls, see Supplementary Figures 3B, 4D. Images were analyzed using ImageJ. In additional lung sections, cell death after LPS challenge was detected by a terminal deoxynucleotidyl transferase dUTP nick end-labeling (TUNEL) assay according to the manufacturer's instructions (4812-30-K; R&D Systems).

### Statistical Analysis

Statistical analysis was performed by using one-way analysis of variance (ANOVA) followed by Bonferroni *post hoc* test or Student's *t*-test to compare two groups (GraphPad Software; Version 7.03; San Diego). Data are presented as the mean ± SEM unless otherwise indicated. Statistical significance was labeled as ^*^p < 0.05; ^**^p < 0.01; ^***^p < 0.001; and ^****^p < 0.0001.

## Results

### CX_3_CL1 Is Produced During Pulmonary Inflammation

**Figure 1:**
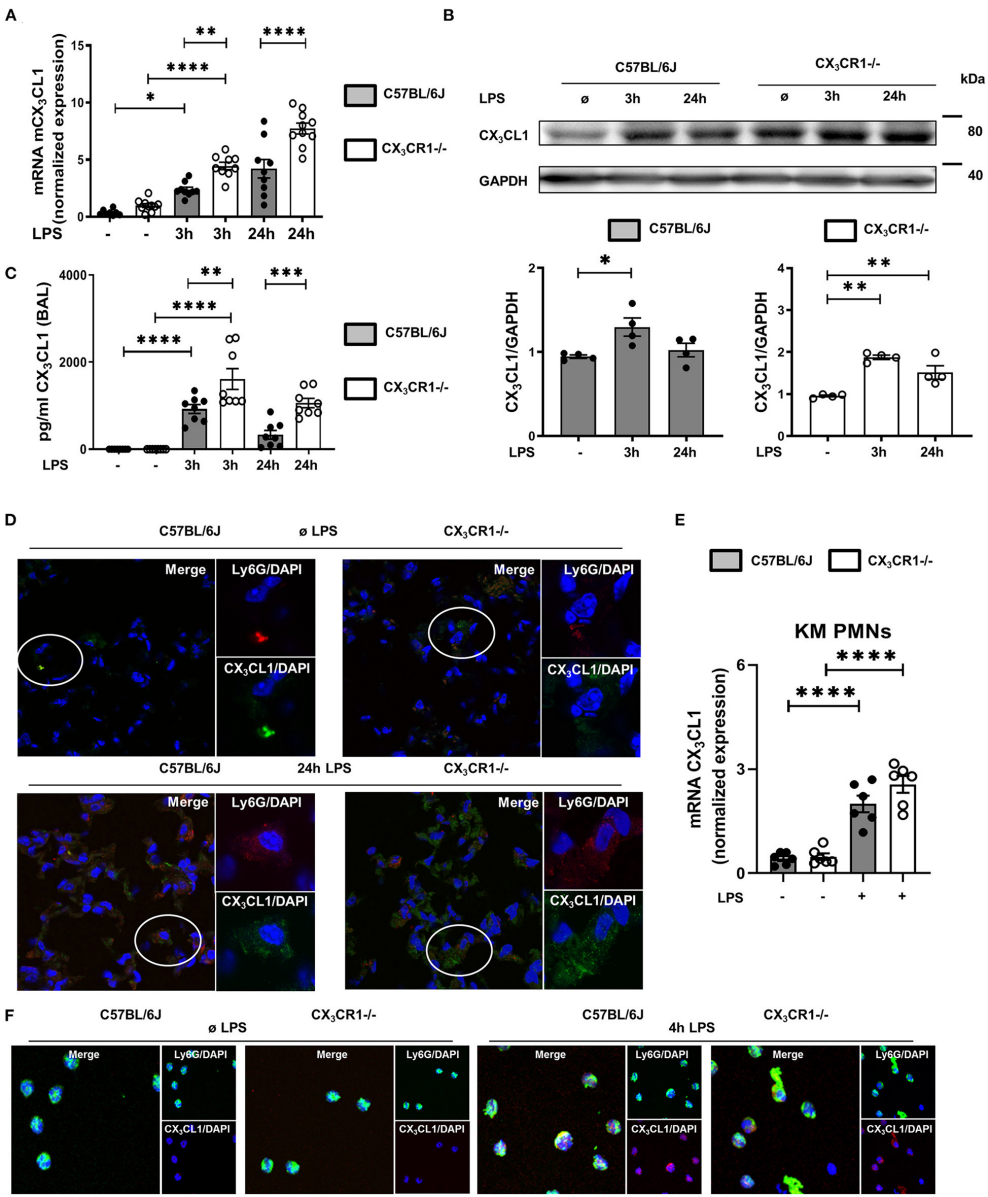
CX_3_CL1-CX_3_CR1 axis during pulmonary inflammation. Three and 24 h after LPS inhalation, the gene **(A)** and protein **(B)** expression of CX_3_CL1 was detected in the pulmonary tissues of C57BL/6J and CX_3_CR1^−/−^ mice (**A**: *n* = 8–10; **B**: representative blots from 3 independent experiments; *n* = 3). The intensity of the blots was evaluated by ImageJ. **(C)** CX_3_CL1 release was determined in the alveolar space of C57BL/6J and CX_3_CR1^−/−^ mice at the indicated time points (*n* = 8). **(D)** Immunofluorescence staining of fractalkine (green) in murine PMNs (red) after LPS in the lungs of C57BL/6J and CX_3_CR1^−/−^ mice (original magnification 40x; one representative image of four is shown; *n* = 4). CX_3_CL1 expression in the bone marrow PMNs in the C57BL/6J and CX_3_CR1^−/−^ mice after LPS exposure (*n* = 6) was determined by RT-PCR **(E)** and immunofluorescence **(F)** (CX_3_CL1: red; murine PMNs: green; original magnification 63x; one representative image of four is shown; *n* = 4). All the data are presented as the mean ± SEM; *p < 0.05; **p < 0.01; ***p < 0.001; ****p < 0.0001; one-way ANOVA + Bonferroni test.

In a murine model of LPS-induced acute pulmonary inflammation, we investigated the role of the chemokine CX_3_CL1 and its receptor, CX_3_CR1. LPS caused a significant increase in CX_3_CL1 expression in the lungs of wild-type (C57BL/6J) and CX_3_CR1^−/−^ mice (Figure 1A). In addition, CX_3_CL1 expression was significantly higher in the CX_3_CR1^−/−^ mice than in the C57BL/6J mice. The protein levels of CX_3_CL1 in the lungs of the C57BL/6J and CX_3_CR1^−/−^ mice confirmed the gene expression analysis results (Figure 1B). Additionally, the release of CX_3_CL1 into the alveolar space of the C57BL/6J mice was significantly increased after LPS administration and was significantly higher in the CX_3_CR1^−/−^ mice (Figure 1C). Immunofluorescence staining further revealed an increase in CX_3_CL1 surface expression on the PMNs in the lungs of the C57BL/6J and CX_3_CR1^−/−^ mice after the onset of inflammation (Figure 1D). To verify this finding, we evaluated the expression of fractalkine on isolated murine bone marrow PMNs from C57BL/6J and CX_3_CR1^−/−^ mice after LPS stimulation and observed a significant increase in the CX_3_CL1 mRNA (Figure 1E) and protein levels in both strains (Figure 1F).

### Depletion of CX_3_CR1 Affects the Inflammatory Response During Pulmonary Inflammation

**Figure 2:**
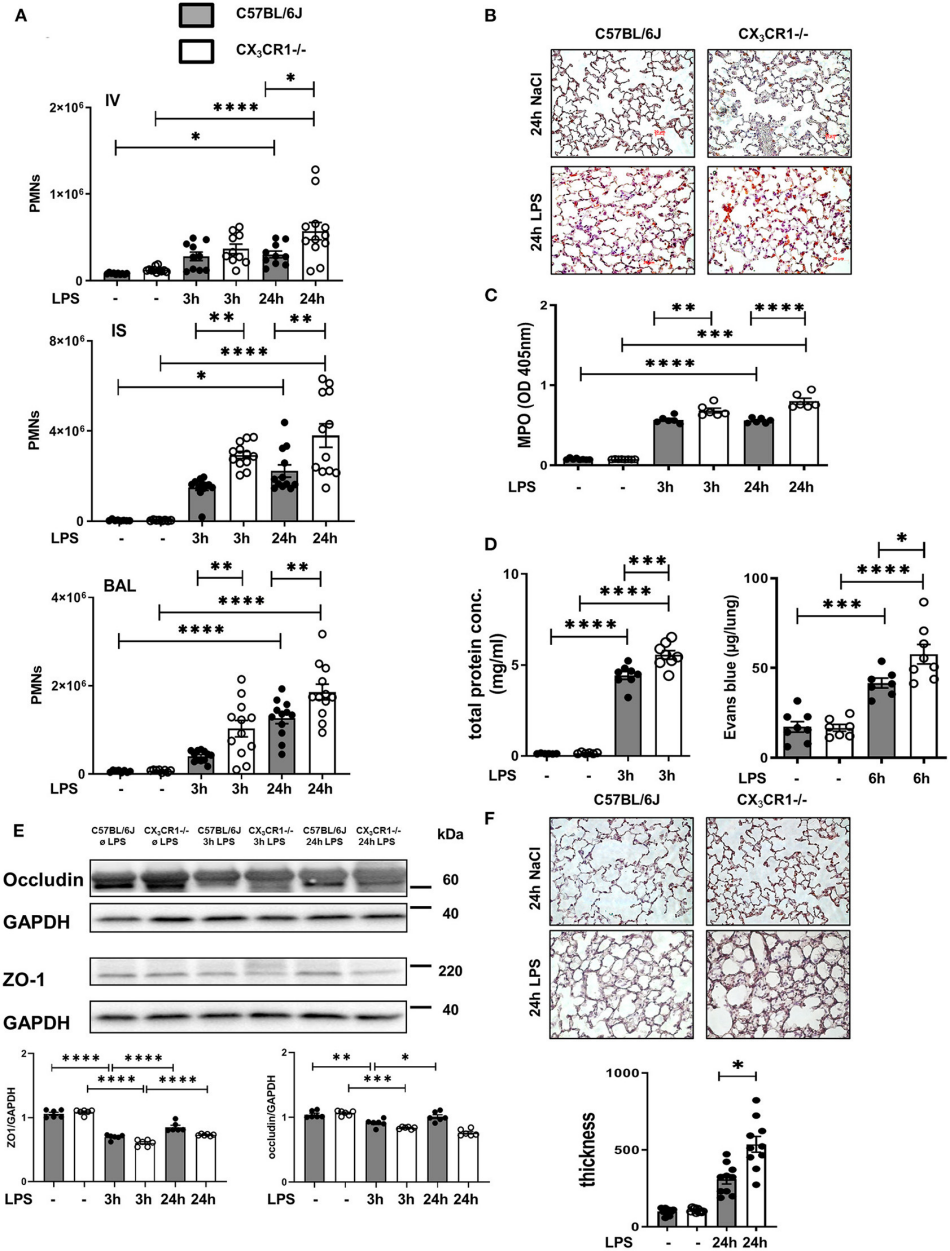
CX_3_CR1 deficiency affects neutrophil transmigration and permeability *in vivo*. **(A)** Flow cytometry-based quantification of intravascular (IV)-, interstitial (IS)-, and bronchoalveolar (BAL)-localized polymorphonuclear neutrophils (PMNs) in C57BL/6J (black filled bar) and CX_3_CR1^−/−^ (open bar) mice after LPS inhalation (*n* = 8–12). **(B)** Quantification of PMN infiltration into the lungs of C57BL/6J and CX_3_CR1^−/−^ mice by immunohistochemistry. PMNs were stained with a specific marker (rat anti-mouse neutrophil, clone RB6-8C5) and appeared brown (original magnification 40x; one representative image is shown; *n* = 4). **(C)** Myeloperoxidase (MPO) was measured in the alveolar lavage of C57BL/6J and CX_3_CR1^−/−^ mice (*n* = 6–8). **(D)** The effect of CX_3_CR1 on microvascular leakage was assessed by evaluation of protein extravasation in the bronchoalveolar fluid of C57BL/6J and CX_3_CR1^−/−^ mice (*n* = 6–8). In separate experiments, Evans blue extravasation was evaluated (*n* = 6–8). **(E)** Tight junction proteins zona occludin (ZO)-1 and occludin were measured in the lung samples of C57BL/6J and CX_3_CR1^−/−^ mice by immunoblots at the indicated times after LPS inhalation (representative blots from 3 independent experiments; *n* = 3). The intensity of the blots was evaluated by ImageJ. **(F)** Evaluation of septal thickness in the lung sections stained with hematoxylin (original magnification 40x; one representative image of four is shown; *n* = 4). All the data are presented as the mean ± SEM; *p < 0.05; **p < 0.01; ***p < 0.001; ****p < 0.0001; one-way ANOVA + Bonferroni test.

We quantified the accumulation of endothelial adherent, interstitial and intra-alveolar PMNs in the C57BL/6J and CX_3_CR1^−/−^ mice by a flow cytometry-based method (Figure 2A). Inflammation significantly increased the PMN counts in all three compartments of the lung in both mouse strains. Therefore, the PMN influx into the lung interstitium and alveolar space in the early (3 h after LPS stimulation) and late (24 h after LPS stimulation) phases of acute inflammation was significantly higher in the CX_3_CR1^−/−^ mice compared to the C57BL/6J mice. This finding was further confirmed by immunohistochemistry, where PMNs were labeled brown to visualize their infiltration into the inflamed lung tissue. After LPS exposure, we quantitatively observed more PMNs in the CX_3_CR1^−/−^ lung tissue than in the wild-type tissue (Figure 2B, Supplementary Figure 4A). To further verify our findings, we determined the concentration of myeloperoxidase (MPO) in alveolar lavage. Myeloperoxidase is an enzyme that is released from PMNs after their activation (21)]. Significantly higher levels of MPO were found in the CX_3_CR1^−/−^ mice compared to the C57BL/6J mice, confirming our previous findings from flow cytometry and immunochemistry (Figure 2C). In addition to PMN migration, the second hallmark of acute pulmonary inflammation is the increase in microvascular permeability, which was assessed by the determination of protein accumulation in the alveolar space and Evans blue extravasation in the lung tissue. Inflammation increased the capillary leakage in both mouse strains, whereas the CX_3_CR1^−/−^ animals showed a significantly higher increase in the protein levels and Evans blue extravasation (Figure 2D), indicating a pivotal role of CX_3_CR1 in microvascular permeability. To further determine the impact of CX_3_CR1 on pulmonary barrier function, we evaluated the effect of this receptor on the tight junction proteins zona occludens (ZO)-1 and occludin (Figure 2E). Occludin interacts with ZO-1 in the cytoplasm and stabilizes endothelial barrier function (22, 23). Representative blots demonstrated a reduction in ZO-1 and occludin after LPS inhalation. In the CX_3_CR1^−/−^ mice, the levels of both tight junction proteins were lower than those in the C57BL/6J mice, confirming and especially explaining our previous findings regarding the detrimental role of CX_3_CR1 in endothelial integrity. To visualize the impact of CX_3_CR1 on tissue damage and altered alveolar walls, we examined HE-stained lung sections and measured septal thickness. CX_3_CR1 depletion caused a significant increase in the alveolar thickness, further emphasizing our previous findings (Figure 2F).

### Loss of CX_3_CR1 Increases the Amount of Dead PMNs in the Lung

**Figure 3:**
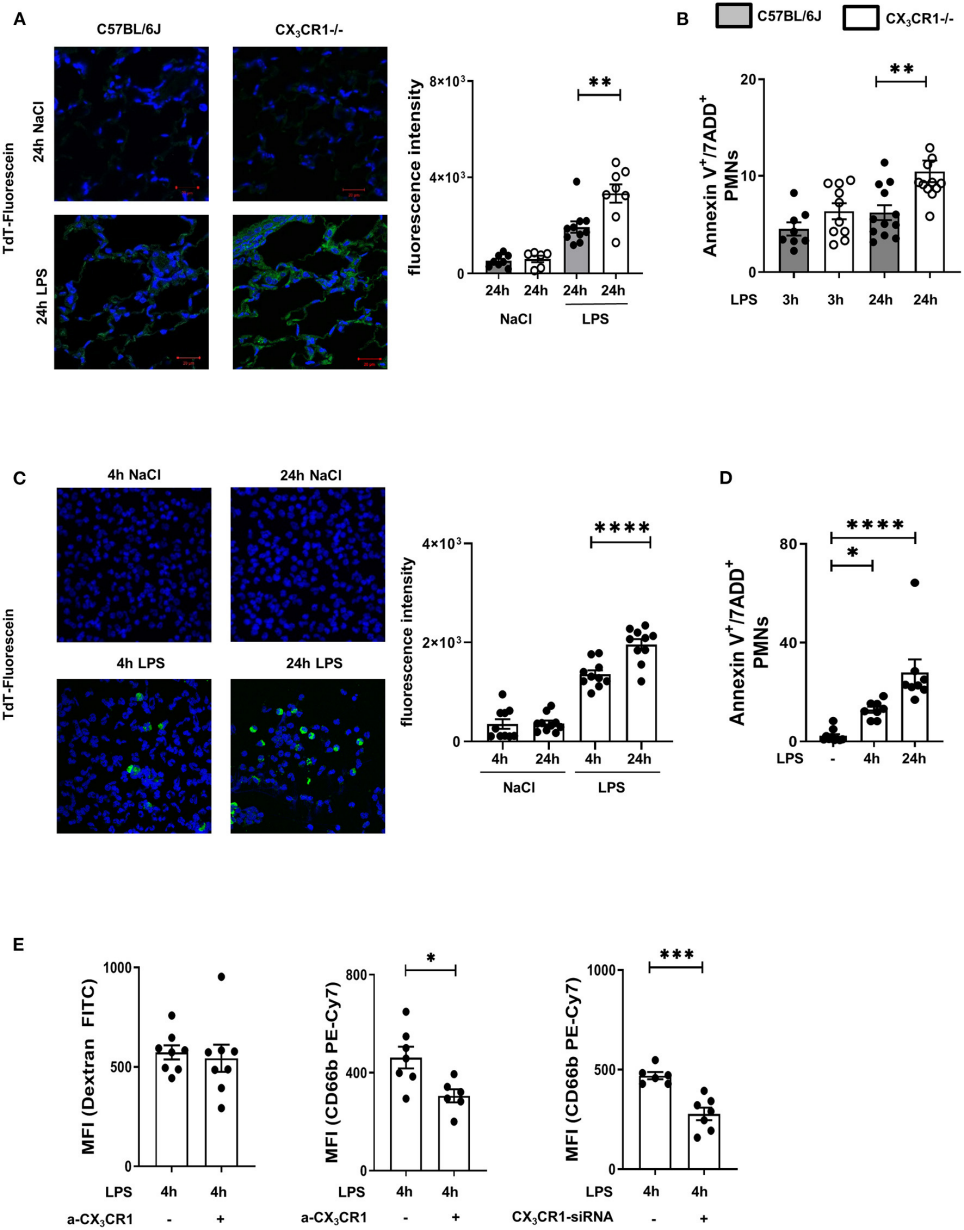
Increased accumulation of dead PMNs in CX_3_CR1^−/−^ mice. **(A)** Fluorescein TUNEL staining in the pulmonary tissue of C57BL/6J and CX_3_CR1^−/−^ mice 24 h after NaCl or LPS exposure. Dead cells were stained with fluorescein green (original magnification 63x, one representative image is shown; *n* = 4), and the fluorescence intensity was assessed (*n* = 8–10). **(B)** Necroptotic/late apoptotic PMNs in the lung interstitium were assessed by Annexin V positive staining. 7-ADD staining was used to identify the necroptotic/late apoptotic cells in C57BL/6J and CX_3_CR1^−/−^ mice 3 and 24 h after LPS inhalation (*n* = 8–12). **(C)** Dead human PMNs were identified by using TUNEL staining 4 and 24 h after NaCl or LPS exposure (original magnification 63x; one representative image of six is shown; *n* = 4) and were stained with fluorescein green, and the fluorescence intensity was evaluated (*n* = 10). **(D)** Isolated human PMNs were stimulated with LPS, and *in vitro* necroptosis/late apoptosis was evaluated by Annexin V/7ADD staining (*n* = 8–10). **(E)** Effects of pharmacological inhibition (a-CX_3_CR1) and gene silencing (siRNA) of CX_3_CR1 on the clearance of FITC-conjugated dextran and human PMNs by human macrophages/monocytes were determined (*n* = 6–8). All the data are presented as the mean ± SEM; *p < 0.05; **p < 0.01; ***p < 0.001; ****p < 0.0001; statistical analyses were performed by Student's *t*-test to compare two groups or one-way ANOVA + Bonferroni test to compare multiple groups.

A common way to identify dead cells is TUNEL fluorescein staining (24). We observed a significant accumulation of dead cells in the CX_3_CR1^−/−^ lung tissue compared to the wild-type lung tissue (Figure 3A; Supplementary Figure 4E). Further investigations of the migrated PMNs in the lung interstitium revealed a significant increase in necroptotic/late apoptotic PMNs in the CX_3_CR1^−/−^ mice compared to those in the C57BL/6J mice (Figure 3B) 24 h after LPS inhalation.

To verify the effects of the CX_3_CL1-CX_3_CR1 axis on necroptotic/late apoptotic human PMNs, we isolated PMNs from healthy volunteers and activated them with LPS. Dead PMNs were identified by TUNEL fluorescein staining (Figure 3C), and the increase in TdT fluorescein intensity indicated increased PMN death over time. To confirm this finding, we additionally identified necroptotic/late apoptotic PMNs by flow cytometry and observed a significant increase (Figure 3D). We incubated these LPS-induced necroptotic/late apoptotic PMNs with a monocyte/macrophage cell line. These monocyte/macrophage cells were treated with a specific inhibitor of CX_3_CR1, and the receptor was depleted by siRNA (Figure 3E). The non-specific clearance capacity of the macrophages was assessed by phagocytosis of FITC-conjugated dextran, and the results showed that this capacity was not impaired by specific CX_3_CR1 inhibition, indicating that CX_3_CR1 inhibition does not influence the general phagocytosis capacity of macrophages. After inhibiting CX_3_CR1, the clearance of necroptotic/late apoptotic PMNs by the MM6 cells was significantly reduced compared to that of the MM6 cells. Depletion of CX_3_CR1 by siRNA also reduced PMN clearance, confirming our results and highlighting the effect of CX_3_CR1 on the removal of dead PMNs. Since late apoptotic and necroptotic PMNs cannot be distinguished *via* flow cytometry or TUNEL staining (10) and the CX_3_CR1^−/−^ mice were clearly more compromised by acute inflammation, we determined the gene expression of the necrosome-associated kinases RIPK1 and RIPK3 in the LPS-inflamed lungs.

### Depletion of CX_3_CR1 Exacerbates Necrosome Activation

**Figure 4:**
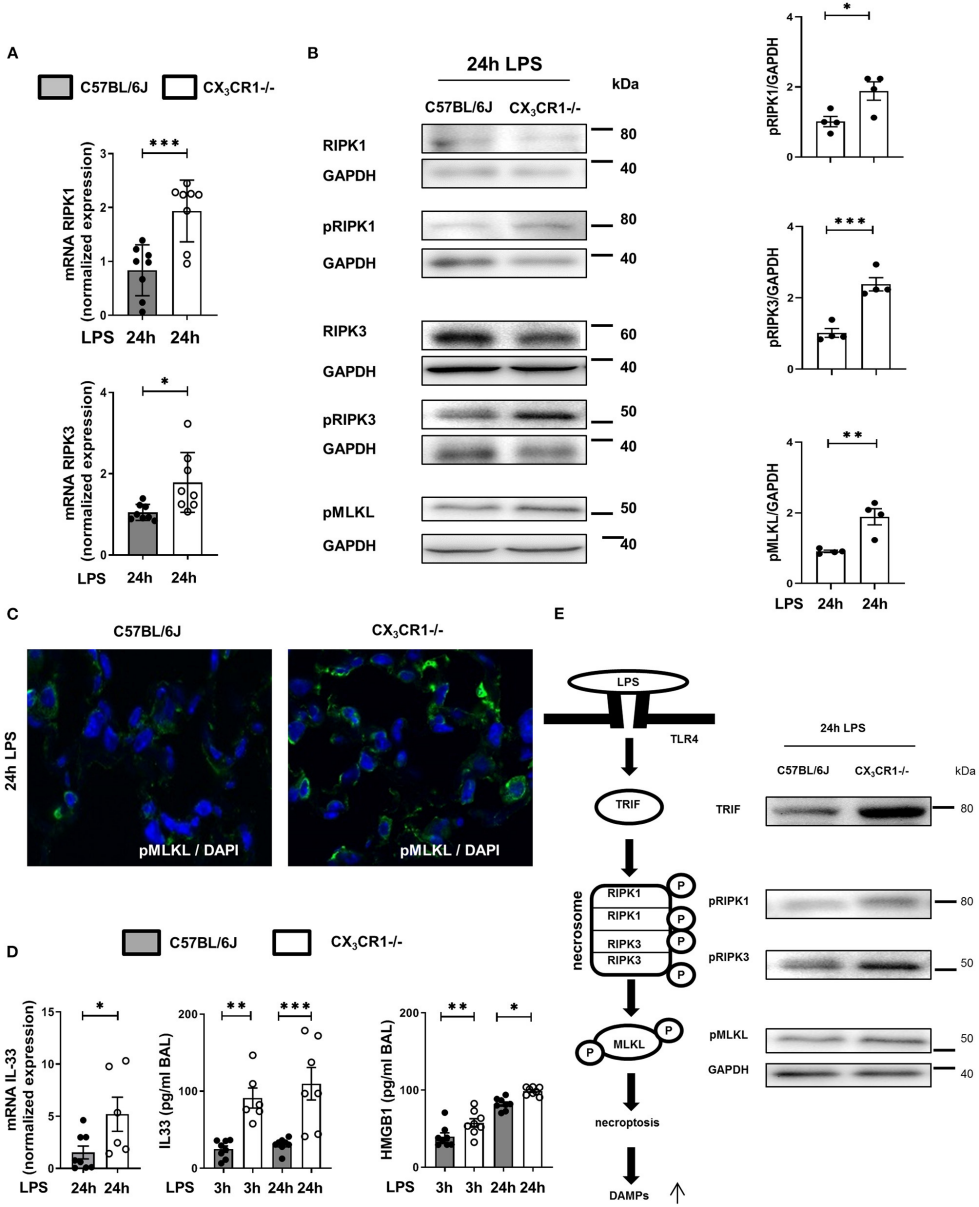
*In vivo* detection of necrosome activation after the onset of inflammation. Expression of the necrosome-related receptor-interacting serine/threonine-protein kinase (RIPK)1 and RIPK3 at the gene **(A)** (*n* = 8) and protein **(B)** levels was detected in the lungs of C57BL/6J and CX_3_CR1^−/−^ mice 24 h after LPS exposure. The protein expression was further analyzed by measuring the phosphorylation and therefore activation of RIPK1, RIPK3, and mixed lineage kinase domain-like protein (MLKL). Representative blots are shown, and the density was assessed (*n* = 3–4). The intensity of the blots was evaluated by ImageJ. **(C)** The phosphorylated form of MLKL was also detected by immunofluorescence in the lungs of C57BL/6J and CX_3_CR1^−/−^ mice (pMLKL appears green; original magnification 63x; one representative image of six is shown; *n* = 4). **(D)** Gene expression of IL-33 was determined in the lung tissue of C57BL/6J and CX_3_CR1^−/−^ mice 24 h after LPS exposure (*n* = 6–8). The release of IL-33 and HMGB1 was evaluated in the bronchoalveolar lavage of C57BL/6J and CX_3_CR1^−/−^ mice (*n* = 6–8). **(E)** Schematic diagram of LPS-dependent necrosome formation in murine lungs. Transmembrane toll-like receptor (TLR) 4 activates (toll/interleukin-1 receptor) domain-containing adaptor protein (TRIF), leading to the phosphorylation of RIPK1 and RIPK3. Necrosome activation induces the phosphorylation of MLKL, resulting in necroptosis and the release of damage-associated patterns (DAMPs). All the data are presented as the mean ± SEM; *p < 0.05; **p < 0.01; ***p < 0.001; statistical analyses were performed by Student's *t*-test to compare two groups or one-way ANOVA + Bonferroni test to compare multiple groups.

The gene expression of RIPK1 and RIPK3 was significantly increased in the CX_3_CR1^−/−^ mice compared to the C57BL/6J mice (Figure 4A). Next, we evaluated the levels of the total and phosphorylated RIPK1 and RIPK3 proteins. The activated form of these kinases leads to the activation of MLKL, which induces necroptosis. The phosphorylation of RIPK1, RIPK3, and MLKL was increased in the CX_3_CR1^−/−^ mice compared to the C57BL/6J mice (Figure 4B). We confirmed these findings by immunofluorescence staining, and the activated form of MLKL in the inflamed lung tissue of the CX_3_CR1^−/−^ mice was also enhanced compared to that of the C57BL/6J mice (Figure 4C). MLKL-mediated necroptosis leads to the release of so-called alarmins (IL-33) and DAMPs (HMGB1). We detected an increase in the gene expression of IL-33 and an aggravated release of IL-33 and HMGB1 in bronchoalveolar lavage (BAL) from the CX_3_CR1^−/−^ mice compared to that from the C57BL/6J mice (Figure 4D). Taken together, we revealed a detrimental role of CX_3_CR1 in the LPS-mediated activation of necrosomes. Toll-like receptor (TLR) 4 signaling induces the tumor necrosis factor receptor-associated factor (TRAF) family. The TRAF family consists of intracellular proteins that act as signaling adaptors, linking upstream receptors to downstream effector enzymes. TRAF2 modulates signaling by death receptors (25), whereas TRAF6 is upstream of TRIF (Toll/interleukin 1 receptor domain-containing adaptor protein inducing interferon beta), which activates RIPK1 and NFκB (26). In the present study, LPS enhanced TRIF *via* TRAF6 but not TRAF2 (Supplementary Figure 3) and promoted the activation of the necrosome-associated RIPK1, RIPK3, and MLKL. This process leads to the necroptosis of PMNs in inflamed lung tissue and the release of DAMPs (Figure 4E). All the signaling proteins were significantly more highly expressed in the CX_3_CR1^−/−^ mice, explaining our previous findings.

### Inhibition of the CX_3_CL1-CX_3_CR1 Axis Exacerbates Inflammation and Increases Necrosome Formation *in vivo*

**Figure 5:**
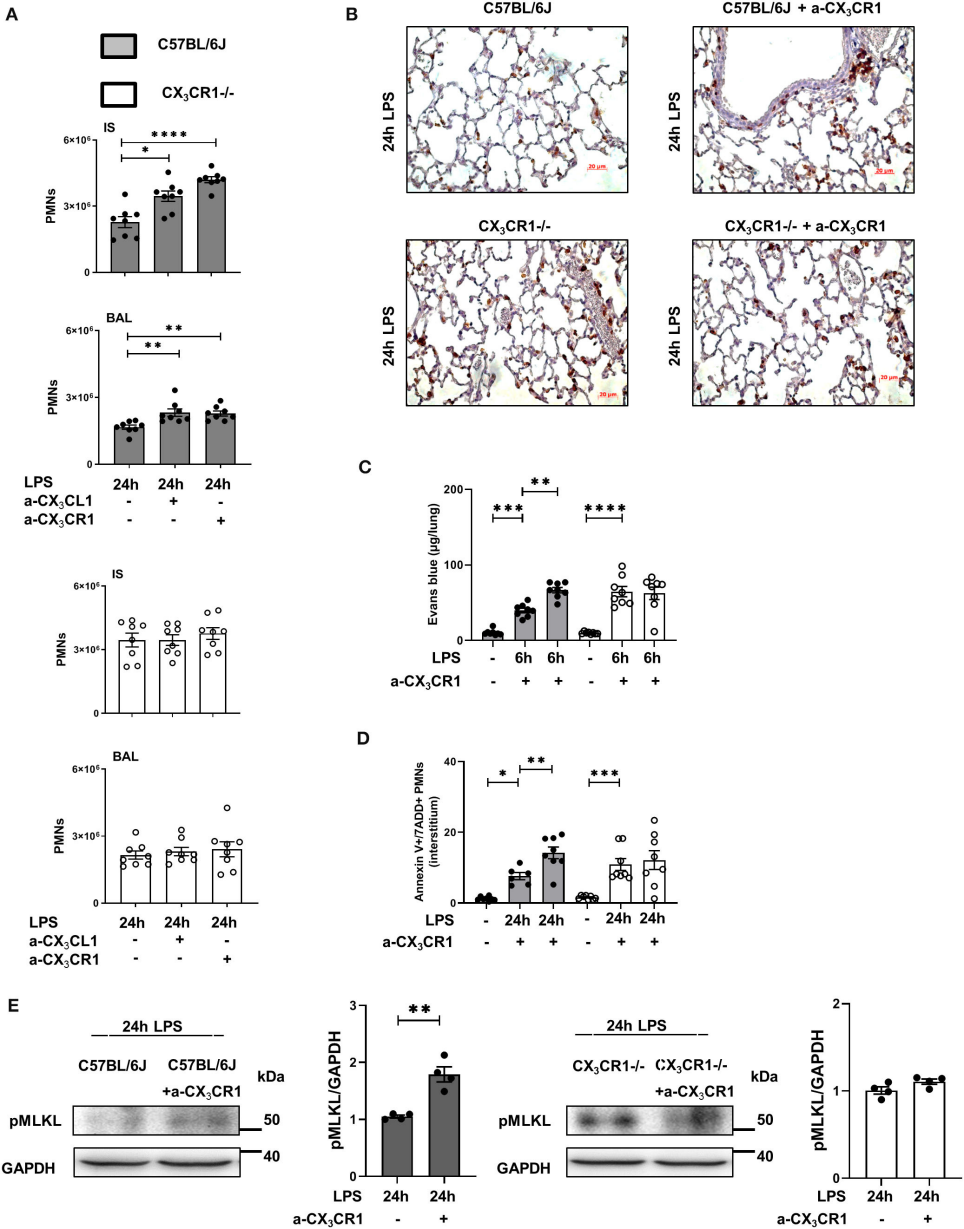
Effects of CX_3_CR1 inhibition on PMN accumulation, microvascular permeability and necrosome formation *in vivo*. **(A)** Twenty-four hours after LPS exposure, the effects of the specific CX_3_CR1 antagonist (TP501) and the specific CX_3_CL1 antagonist (TP233) on the interstitial and alveolar PMN counts in C57BL/6J (filled bar) and CX_3_CR1^−/−^ (open bar) mice (*n* = 8) were evaluated. **(B)** PMN infiltration into the lungs of C57BL/6J and CX_3_CR1^−/−^ mice was also quantified by immunohistochemistry, and the effect of the specific CX_3_CR1 antagonist was determined. PMNs were stained with a specific marker (rat anti-mouse neutrophil; clone RB6-8C5) and appeared brown (original magnification 40x; one representative image of four is shown; *n* = 4). The effects of the specific CX_3_CR1 antagonist on **(C)** Evans blue extravasation in the lung tissue and **(D)** on the fraction of AnnexinV^+^/7ADD^+^ (necroptotic/late apoptotic) PMNs in the interstitium of the lung of C57BL/6J and CX_3_CR1^−/−^ mice (*n* = 8) were evaluated. **(E)** Twenty-four hours after LPS inhalation, the phosphorylated form of MLKL after treatment with the specific CX_3_CR1 antagonist was detected by western blot in the lungs of C57BL/6J and CX_3_CR1^−/−^ mice (representative blots from 3 independent experiments; *n* = 3). The intensity of the blots was evaluated by ImageJ. All the data are presented as the mean ± SEM; *p < 0.05; **p < 0.01; one-way ANOVA + Bonferroni test. ***p < 0.001; ****p < 0.0001.

Blockade of CX_3_CR1 and CX_3_CL1 increased the PMN influx into the lung interstitium and alveolar space in the C57BL/6J mice but had no effects in the CX_3_CR1^−/−^ mice (Figure 5A). These findings excluded the possibility that the increased inflammation in the CX_3_CR1^−/−^ mice is based on increased fractalkine concentrations in these animals (Figure 1). We further confirmed this finding by immunohistochemistry, in which PMNs were labeled brown to visualize their infiltration into the inflamed lung tissue (Figure 5B). Microvascular permeability was increased in the C57BL/6J mice after the specific inhibition of CX_3_CR1, but the capillary leakage in the CX_3_CR1^−/−^ mice was not affected (Figure 5C). Confirming our previous data, the specific antagonism of CX_3_CR1 increased the accumulation of necroptotic/late apoptotic PMNs in the lung interstitium after the onset of inflammation in the C57BL/6J mice (Figure 5D). Specific inhibition of CX_3_CR1 resulted in an augmentation of the phosphorylated form of MLKL in the C57BL/6J animals (Figure 5E).

### Absence of CX_3_CR1 Leads to NFκB-Mediated Release of Inflammatory Cytokines

**Figure 6:**
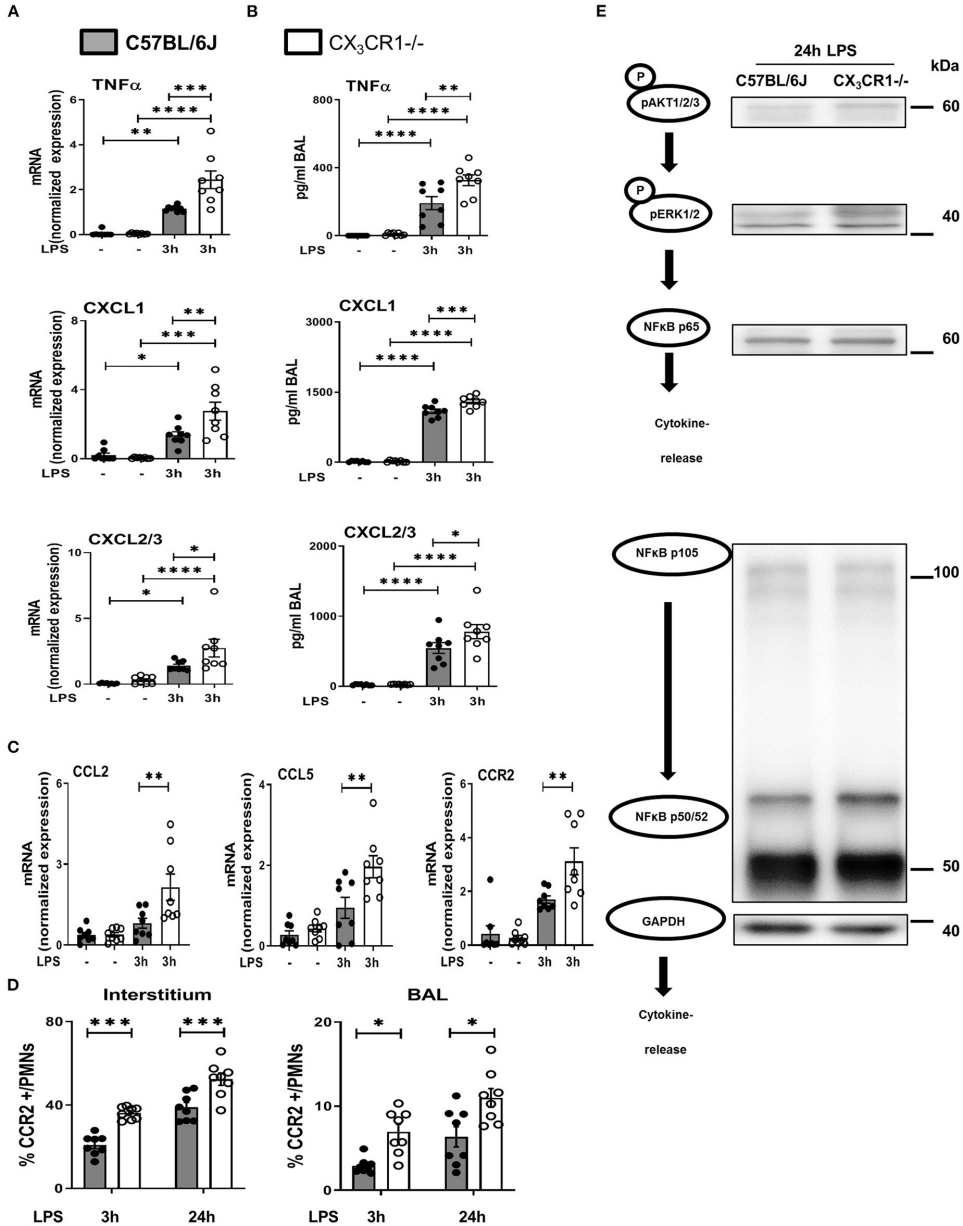
NFκB-dependent release of inflammatory cytokines *in vivo*. Gene **(A)** and protein **(B)** expression of TNFα, CXCL1 and CXCL2/3 was evaluated in the in bronchoalveolar lavage (BAL) of the lungs of C57BL/6J and CX_3_CR1^−/−^ mice (*n* = 8). **(C)** Gene expression of CCL2, CCL5 and CCR2 was detected (*n* = 8), and **(D)** CCR2-positive PMNs in the lung interstitium and alveolar space of C57BL/6J and CX_3_CR1^−/−^ mice were identified (*n* = 8). **(E)** Schematic diagram of the phosphorylated forms of AKT1/2/3, ERK1/2, NFκB p65, NFκB p50/52, NFκB p105 and their effect on the release of inflammatory cytokines (representative blots from 3 independent experiments; *n* = 3). All the data are presented as the mean ± SEM; *p < 0.05; **p < 0.01; ***p < 0.001; ****p < 0.0001; one-way ANOVA + Bonferroni test.

LPS inhalation induced a significant increase in the gene and protein expression of the cytokine TNFα, and this increase was significantly higher in the CX_3_CR1^−/−^ mice (Figures 6A,B). The main PMN chemoattractants CXCL1 and CXCL2/3 were also increased significantly more in the CX_3_CR1^−/−^ mice after the induction of inflammation, confirming our *in vivo* results regarding PMN migration into the different compartments of the lung. Since CX_3_CR1 has been mainly investigated in macrophages to date, we also measured the transcription of CCL2 and CCL5 in murine lungs after the onset of inflammation (Figure 6C). These two chemokines are mainly released by macrophages after NFκB activation and are also critically involved in PMN migration (27). LPS did not increase CCL2 and CCL5 transcription in the C57BL/6J mice, but it was significantly elevated in the CX_3_CR1^−/−^ mice. Concordantly, the expression of CCR2 on PMNs was higher in the interstitium and alveolar space in the CX_3_CR1^−/−^ mice than in the C57BL/6J mice (Figure 6D). Protein analyses further revealed that the absence of CX_3_CR1 leads to an increased expression of phosphorylated AKT1/2/3, resulting in an increased activation of the ERK1/2 and NFκB pathways (p65, p50/52, and p105) (Figure 6E). NFκB induces the transcription of various inflammatory genes, such as inflammatory chemokines and cytokines, thus explaining our previous findings.

### LPS-Mediated Necrosome Formation in Human PMNs

**Figure 7:**
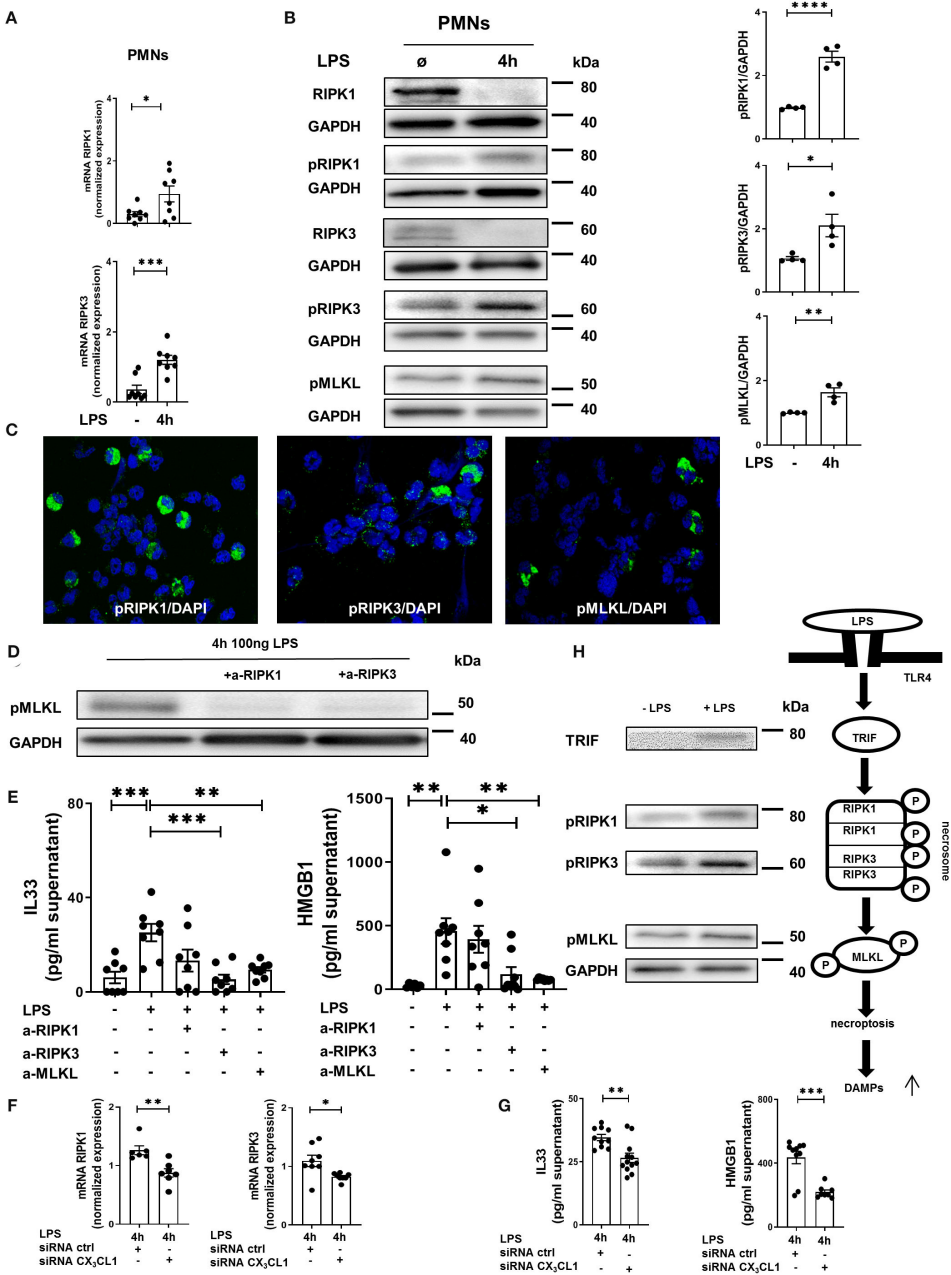
*In vitro* necrosome activation on human PMNs. **(A)** Necrosome-related protein kinase RIPK1 and RIPK3 expression in human PMNs after LPS stimulation (*n* = 8) was detected by RT-PCR. **(B)** Immunoblots of RIPK1, RIPK3, MLKL and their phosphorylated forms were obtained from cell lysates of human PMNs after LPS stimulation (representative blots of *n* = 4–5 are shown). The intensity of the blots was evaluated by ImageJ. **(C)** Phosphorylation of RIPK1, RIPK3, and MLKL was detected in human PMNs after LPS (original magnification 63x; one representative image of five is shown; *n* = 4). **(D)** Effects of specific RIPK1 and RIPK3 inhibition on MLKL phosphorylation in human PMNs after LPS exposure were evaluated (representative blots from 3 independent experiments; *n* = 3). **(E)** Human IL-33 and HMGB1 were determined in the supernatant of stimulated and treated PMNs as indicated (*n* = 6–8). **(F)** Effects of fractalkine depletion on necrosome-related RIPK1 and RIPK3 expression in human PMNs (*n* = 6–8) and on the release of human alarmins IL-33 and HMGB1 **(G)** were evaluated (*n* = 8–12). All the data are presented as the mean ± SEM; *p < 0.05; **p < 0.01; ***p < 0.001; Student's *t*-test to compare two groups or one-way ANOVA + Bonferroni test to compare multiple groups. **(H)** Schematic diagram of LPS-dependent necrosome formation in human PMNs. Transmembrane toll-like receptor (TLR) 4 activates (toll/interleukin-1 receptor) domain-containing adaptor protein (TRIF), leading to the phosphorylation of RIPK1 and RIPK3. Necrosome activation induces the phosphorylation of MLKL, resulting in necroptosis and the release of damage-associated patterns (DAMPs) (representative blots from 3 independent experiments; *n* = 4). ****p < 0.0001.

In human PMNs, inflammation induced a significant increase in the gene expression of the necrosome-associated kinases RIPK1 and RIPK3 (Figure 7A). Western blots revealed a decrease in the total protein levels of RIPK1 and RIPK3 after LPS-induced inflammation but a significant increase in the phosphorylated forms of these necrosome-forming kinases, leading to the activation of MLKL (Figure 7B). We also detected the activated forms of RIPK1, RIPK3, and MLKL in human PMNs after LPS stimulation by immunofluorescence (Figure 7C). The specific inhibition of RIPK1 and RIPK3 resulted in the reduced phosphorylation of MLKL in human PMNs after stimulation with LPS (Figure 7D), further supporting the accuracy of the necrosome cascade. In the supernatant of these stimulated PMNs, LPS also caused a significant increase in IL-33 and HMGB1 (Figure 7E). After the inhibition of RIPK3 and MLKL, both alarmins were significantly reduced. In human PMNs, the specific depletion of fractalkine reduced the gene levels of the necrosome-related RIPK1 and RIPK3 (Figure 7F) and ameliorated the LPS-dependent release of the alarmins IL-33 and HMGB1 (Figure 7G), confirming our *in vivo* results and linking fractalkine to the necrosome complex. We summarized these findings in a diagram of the LPS-mediated activation of RIPK1, RIPK3, and MLKL *via* the activation of TRIF, which leads to necroptosis in human PMNs and the release of DAMPs (Figure 7H).

### Inhibition of MLKL Reduces the Release of Reactive Oxygen Agents by Neutrophils

**Figure 8:**
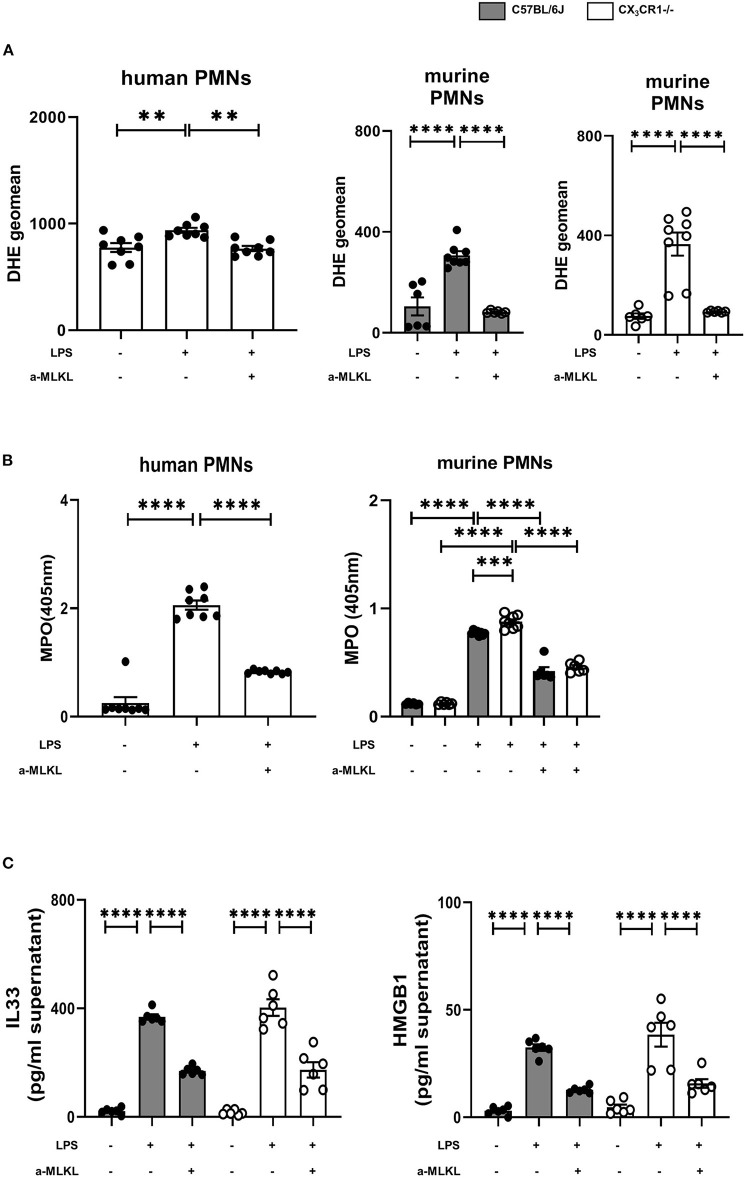
Effects of necrosome-associated proteins on the release of reactive oxygen species (ROS) and myeloperoxidase (MPO) from PMNs. **(A)** The release of ROS was evaluated in human (*n* = 8) and murine bone marrow PMNs after LPS stimulation (*n* = 6–8). Specific MLKL inhibition significantly reduced ROS release. **(B)** LPS-induced MPO formation from human (*n* = 8) and murine bone marrow PMNs was detected (*n* = 6–8). **(C)** MLKL inhibition affects the LPS-induced release of IL-33 and HMGB1 from murine bone marrow PMNs (*n* = 6). All the data are presented as the mean ± SEM; **p < 0.01; ***p < 0.001; ****p < 0.0001; one-way ANOVA + Bonferroni test to compare multiple groups.

The activation of the necrosome complex (RIPK1 and RIPK3) and the subsequent phosphorylation of MLKL has been shown to trigger the downstream production of reactive oxygen species (ROS) (28). Accordingly, we evaluated the correlation of the release of ROS and the activation of the necrosome/fractalkine axis in isolated human and murine neutrophils. Furthermore, we measure the release of MPO, which is a marker of leukocyte activity and a key regulator of oxidative stress that triggers the sensitivity of cells to reactive oxygen products (29). LPS stimulation increased ROS release, but the MLKL inhibitor significantly reduced reactive oxygen species in the human and murine PMNs from both mouse strains (Figure 8A). The release of MPO into the supernatants was diminished after inhibiting MLKL in all the PMNs investigated, further supporting our findings (Figure 8B). As a further marker of cell destruction, we measured gene expression and release of alarmins from murine PMNs (Figure 8C). Inflammation increased the levels of IL-33 and HMGB1 at the protein level. Treatment with the selective MLKL inhibitor decreased the release of alarmins from all the investigated neutrophils.

## Discussion

We determined the role of fractalkine in acute pulmonary inflammation and detected an increase in the gene and protein expression of chemokine in the lungs of mice after the onset of inflammation. We revealed a detrimental role of the CX_3_CR1 receptor in acute pulmonary inflammation; that is, the depletion of the receptor resulted in an increase in chemokines, migrated PMNs and microvascular permeability. To the best of our knowledge, we are the first to show that inflammation increased the chemokine fractalkine in PMNs after the onset of inflammation. Furthermore, we shed light on the fate of the excessively migrated PMNs. In the present study, CX_3_CR1 depletion increased pulmonary inflammation. This finding is consistent with the findings of Medina-Contreras et al., where the knockout of CX_3_CR1 and CX_3_CL1 in mice increased the severity of disease in a colitis model (30); that study examined stool consistency, presence of fecal blood, and weight loss but not the migration of inflammatory cells. In a model of neuroinflammation, CX_3_CR1 deficiency resulted in increased inflammatory chemokines and neurodegeneration (31). In contrast, CX_3_CR1 inhibition decreased cell counts in the BAL and chemokines levels in a model of asthma (17); however, the study was focused on eosinophilia and T cells and therefore is not comparable with the presented results. In a model of bacterial-induced pulmonary inflammation, the authors also observed increased neutrophils in the lungs but did not identify a reason for their findings (32). The fractalkine receptor has mainly been studied in macrophages, where it is used to differentiate between macrophage phenotypes and functions (30, 33–35).

**Figure 9:**
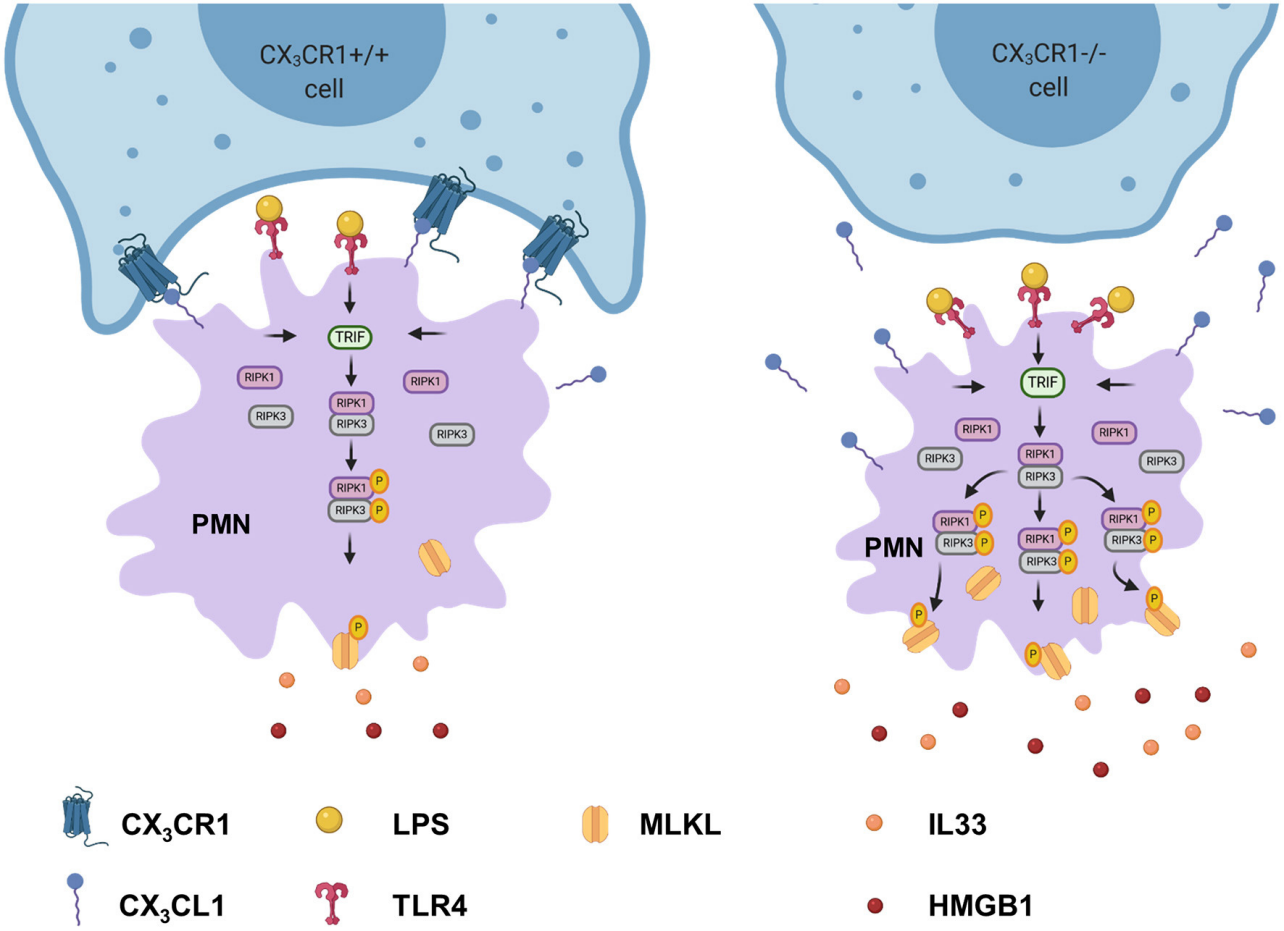
Depletion of CX_3_CR1 leads to increased activation of the necrosome in PMNs. Activation of TLR4 in PMNs by lipopolysaccharide leads to the phosphorylation of the necrosome-related RIPK1, RIPK3, and MLKL. The activated MLKL induces the full necroptotic response by disrupting the cellular membrane, releasing DAMPs (IL-33 and HMGB1) and therefore further increasing inflammation.

We investigated the impact of the receptor on PMNs in acute pulmonary inflammation. CX_3_CR1 inhibition resulted in increased numbers of necroptotic and late apoptotic PMNs in the lung interstitium and BAL, respectively, due to reduced clearance. Twenty-four hours after LPS inhalation, we observed the highest PMN accumulation as shown in our previous studies (36, 37). Our data suggests that the elevated PMN numbers in the BAL and lung tissue were associated with raised counts of necroptotic cells. Apoptotic cell death is a weak inducer of inflammation and is the best-known form of cell death. To date, necrotic cell death has been considered to be accidental and uncontrolled. The recently discovered form of necrotic cell death, “necroptosis” (28, 38), is considered to be pro-inflammatory cell death and is associated with pathological cell injury (39, 40). The so-called necrosome complex consists of the activation of the RIPK1 and RIPK3 protein complex, leading to the phosphorylation and therefore activation of MLKL (Figure 9). MLKL is translocated to the plasma membrane, mediating its permeabilization and DAMP release. DAMPs that are released by the necrosome include HMGB1 and alarmins, which are molecules that perform cytokine-like functions (e.g., IL-1α and IL-33). In the present study, we demonstrated the increased activation of the necrosome and the consequent release of HMGB1 and IL-33 in the CX_3_CR1^−/−^ animals and in the C57BL/6J animals after inhibiting CX_3_CR1. Accordingly, we linked, for the first time, the lack of CX_3_CR1 to the increased activation of the necrosome complex, which is a massive trigger of inflammation (41). TLR4-induced necroptosis critically depends on the activation of TRIF (42), which was significantly higher in the CX_3_CR1^−/−^ animals and in the human PMNs after stimulation with LPS in the present study. TRIF can directly activate RIPK3 independently of RIPK1 (42). Recent literature suggests that RIPK3 is the essential regulatory kinase in this process (43, 44). Nevertheless, our results show a decrease in the total amount of the RIPK1 protein and an increase in the phosphorylated form. Furthermore, inhibiting RIPK1 in human PMNs decreased the phosphorylated form of MLKL, indicating a distinct role of RIPK1 in this setting. On the other hand, only inhibiting RIPK3 decreased IL-33 and HMGB1 to baseline levels after acute inflammation. Additionally, RIPK3 has been shown to be involved in NFκB activation and the resulting inflammatory cytokine release in a model of intestinal inflammation (13, 45).

In our model of acute pulmonary inflammation, we also detected increased chemokine levels in the CX_3_CR1^−/−^ mice based on stronger activation of the AKT, ERK1/2, and NFκB signaling cascades. Strong NFκB activation increases the CCL5 levels (46), confirming the results of our study. Furthermore, Toll-like receptor (TLR)-mediated CCR2 expression in neutrophils is NFκB-dependent (47). In the present study, the gene expression of CCL2, CCL5, CCR2, and the numbers of the CCR2-positive PMNs in the interstitium and BAL increased significantly more in the CX_3_CR1^−/−^ mice than in the C57BL/6J mice after the onset of inflammation. CCR2 has been identified as a receptor that is responsible for the inappropriate recruitment of PMNs to different organs during acute sepsis (47). Similarly, HMGB1 itself further activates NFκB (48) and induces the synthesis of pro-inflammatory cytokines (49), continuing to enhance inflammation. Several publications suggest that RIPK3-induced necroptosis is at least partly due to excessive reactive oxygen production (28, 38, 42). Therefore, it is not clear whether ROS initiate or execute neutrophil necroptosis (50). Zhang et al., in a model of ischemia-reperfusion injury of the heart, showed that that ROS production was significantly lower in the hearts of RIPK3^−/−^ animals (51). Similarly, the release of reactive oxygen species was significantly reduced after MLKL inhibition in the present study. MLKL is the downstream target of RIPK3. To date, there are only a few studies of necroptosis in neutrophils. Wicki et al. demonstrated that mouse neutrophils secrete TNFα, and after inhibiting apoptosis, necroptosis occurs (52). Unlike our study, Wicki et al. used myeloid progenitor cells to generate neutrophils for their experiments. We used mature neutrophils for our experiments, and LPS-induced necroptosis occurred without inhibiting apoptosis. He et al. demonstrated RIPK3-induced necroptosis in mouse macrophages after TLR4 stimulation by LPS, supporting our findings (42). Another group stimulated human PMNs with GM-CSF followed by the ligation of adhesion molecules and therefore mimicked inflammation (53). These authors observed RIPK3- and MLKL-induced necroptosis with high levels of reactive oxygen species. A crucial step in limiting inflammation is the clearance of dying cells (54). Pasparakis and Vandenabeele (40), in their review about the role of necroptosis in inflammation, called for more studies about the regulation of cell death and the subsequent triggering of inflammation. Wang et al. called specifically for further studies about neutrophil necroptosis, which could lead to a new treatment option for inflammatory or autoimmune disease (50). Furthermore, the induction of RIPK3 in humans has been shown in non-alcoholic steatohepatitis (55). Phosphorylated MLKL has been detected in human liver biopsy samples from patients with drug-induced liver injury (56). Recently, the MESSI (Molecular Epidemiology of SepsiS in the Intensive care unit) trial, which is a prospective study with 120 patients, suggested a correlation between increased plasma levels of the necrosome-related RIPK3 and the development of ARDS during sepsis (55). Current research suggests that manipulating necroptosis could lead to new therapeutic strategies for acute and chronic inflammation (40, 57).

Our *in vitro* experiments with human PMNs are, to the best of our knowledge, the first to identify LPS-induced necroptosis in human PMNs and to further establish the link between fractalkine and the necrosome complex. A limitation of our study is that we only examined the LPS-related necroptosis on acute pulmonary inflammation. Furthermore, it would be interesting to investigate the effects of other pro-inflammatory stimulants or various bacteria on necroptotic PMNs. In summary, we identified the CX_3_CR1 receptor as a crucial receptor in acute pulmonary inflammation that affects the two hallmarks PMNs: migration and microvascular permeability. The depletion of this receptor led to elevated CX_3_CL1 expression and results in the activation of the necrosome complex in the PMNs as the cause of the augmented release of DAMPs (Figure 9). Furthermore, the activated necrosome initiated the AKT, ERK1/2, and NFκB signaling cascades, increasing chemokines and cytokines.

## Data Availability Statement

The original contributions generated for the study are included in the article/Supplementary Materials, further inquiries can be directed to the corresponding author.

## Ethics Statement

The animal study was reviewed and approved by Animal Care and Use Committee of the University of Tuebingen.

## Author Contributions

K-CN, JR, and FK designed research. K-CN and JG-T performed experiments. K-CN, JG-T, and FK analyzed data. JR helped to supervise the project. K-CN and FK wrote the manuscript. All authors approved the final manuscript.

## Conflict of Interest

The authors declare that the research was conducted in the absence of any commercial or financial relationships that could be construed as a potential conflict of interest.
