# Supplementary material for: Fractalkine Is Linked to the Necrosome Pathway in Acute Pulmonary Inflammation
Source: Front Med (Lausanne). 2021 Mar 12;8:591790. doi: 10.3389/fmed.2021.591790 (PMC8006293; doi:10.3389/fmed.2021.591790)
Supplement: Supplementary file 1 [file Data_Sheet_1.pdf]

Supplemental Figure 1

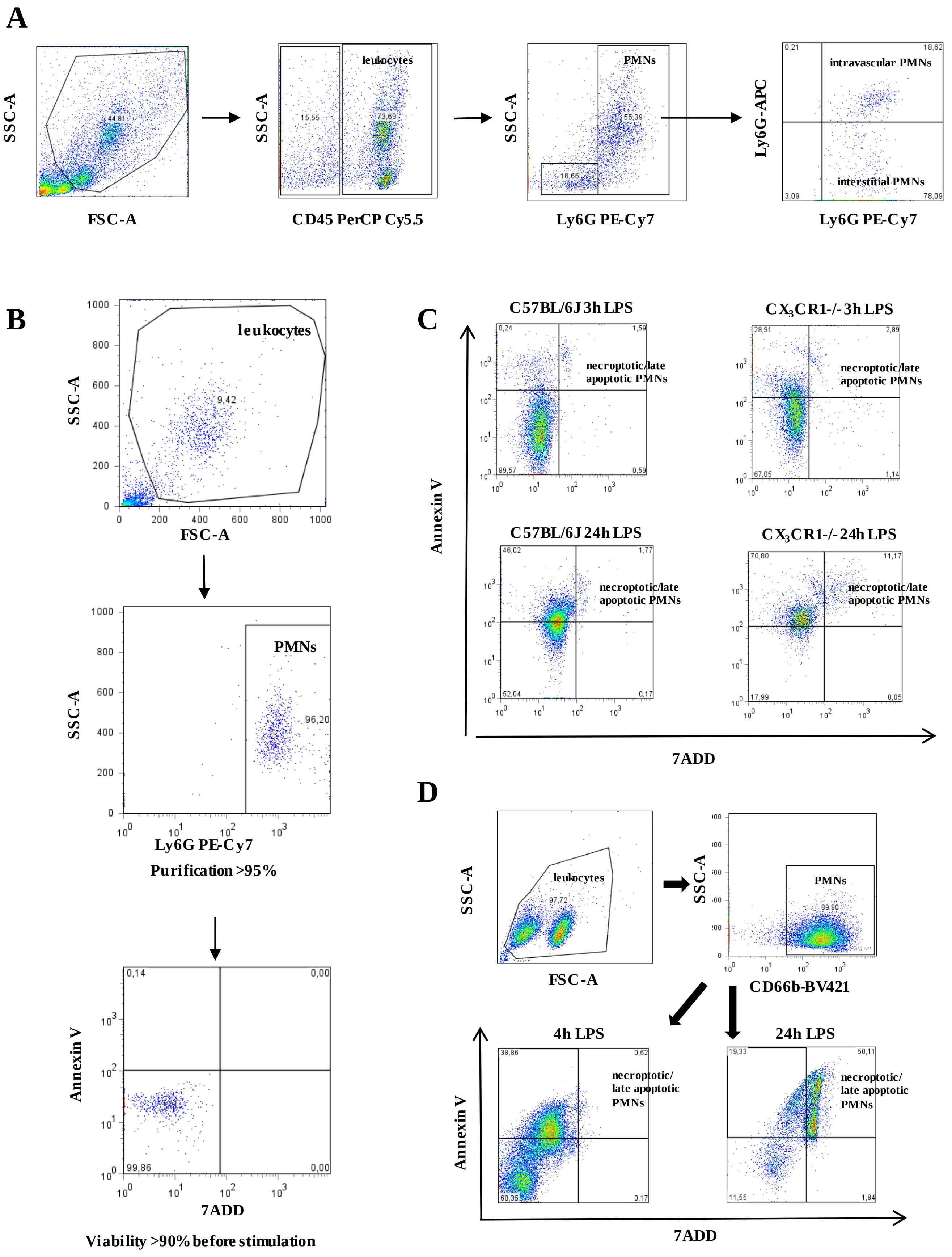

Supplemental figure 1. A) Gating process for the determination of PMNs in lung tissue. CD45 positive cells were gated as leukocytes. PMNs adherent to the pulmonary endothelium are presented as Ly6G APC positive/Ly6G PE-Cy7 positive cells and interstitial PMNs distributed as Ly6G APC negative and Ly6G-PE-Cy7 positive leukocytes. **B)** Purification and viability of bone marrow PMNs were evaluated by a flow cytometry-based method. Leukocytes were gated by typical forward scatter-side scatter (FSC/SSC) appearance and PMNs were tackled by Ly6G-PE/Cy7 and by Annexin V/7ADD-Assay. From leukocytes gate, we identified PMNs by gating Ly6G-PE/Cy7 positive cells. Ly6G-PE/Cy7-positive cells were double stained by Annexin V and 7-ADD to evaluate the viability. We identified the viable cells in the lower left square. **C)** Necroptotic/late apoptotic PMNs were determined from the interstitium of WT- and CX_3CR1-/- animals 3 respectively 24 hours by double staining with Annexin V and 7-ADD (upper right square). **D)** Isolated human PMNs were identified by their typical appearance in FSC/SSC and by CD66b surface expression. Subsequently, PMNs were tackled by Annexin V and 7-ADD double staining and necroptotic/late apoptotic PMNs 4 respectively 24 hours after LPS exposure were gated in the upper right square.

Supplemental Figure 2

A

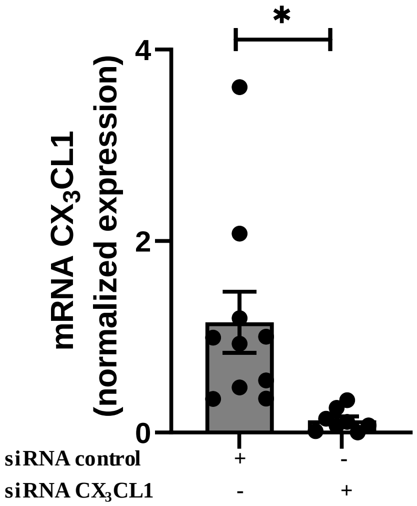

B

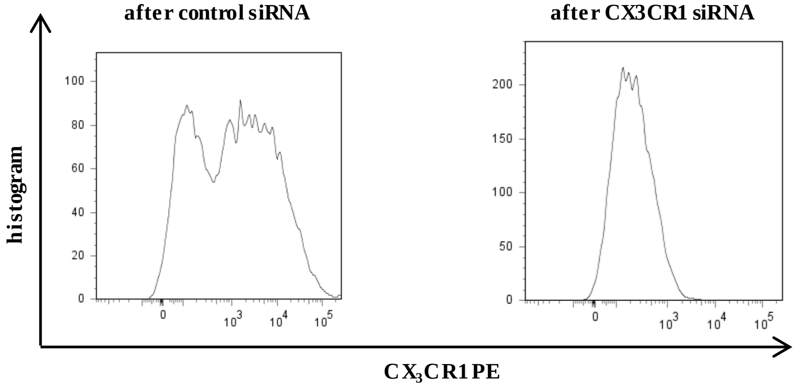

Supplemental figure 2. A) Gene levels of CX_3CL1 after deletion by siRNA CX_3CL1 (n= 7–8). **B)** CX_3CR1 surface expression on MM6 cells were detected after genetic depletion with siRNA CX_3CR1 respectively siRNA control by flow cytometry (n=4).

Supplemental Figure 3

A

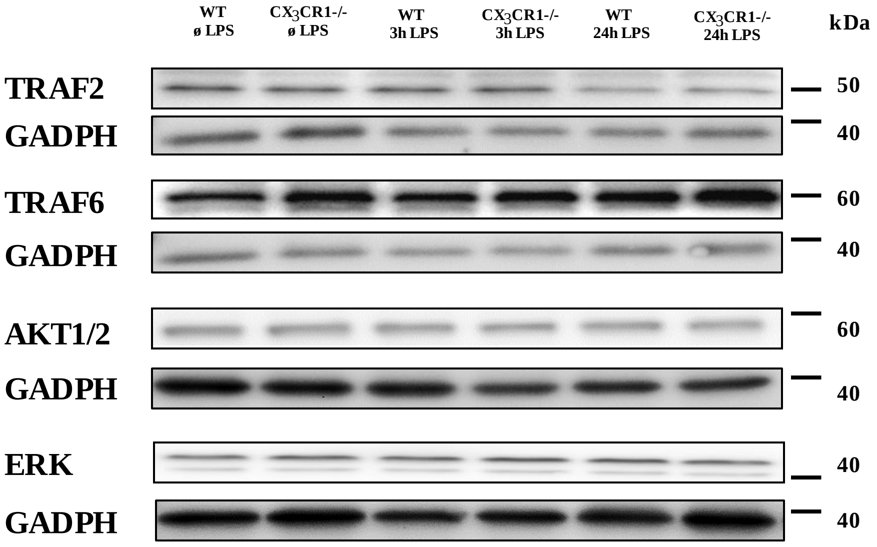

B

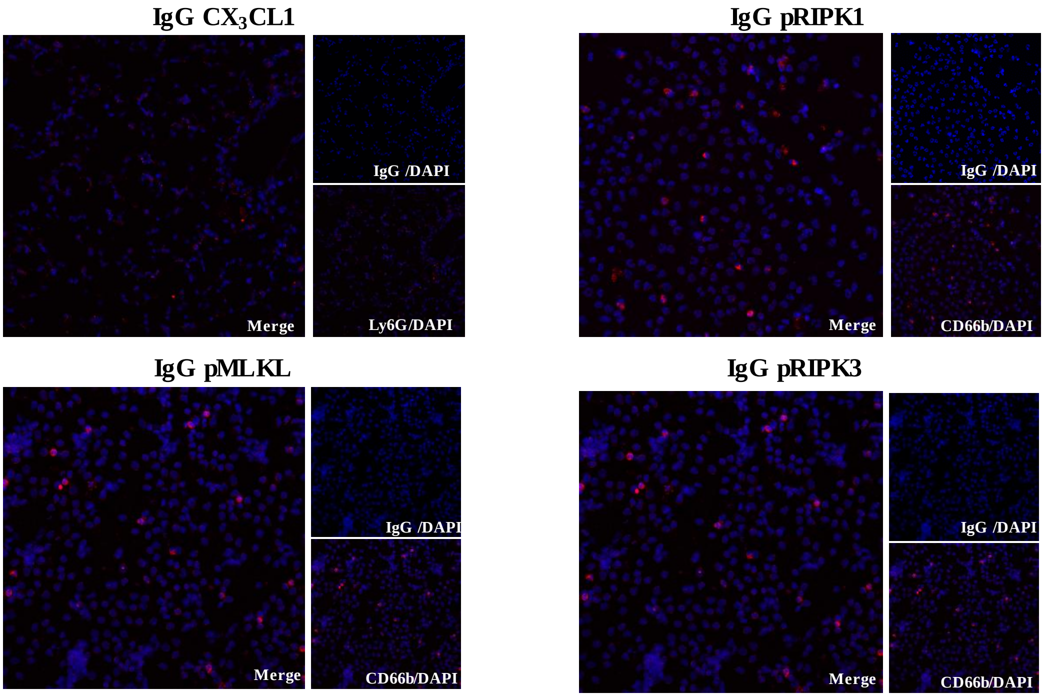

Supplemental figure 3. A) Immunoblots evaluated TRAF2, TRAF6, AKT1/2 and ERK1 expression in lung tissue of WT- and CX_3CR1-/- mice at indicated times after LPS inhalation (representative blots form 3 independent experiments; n=3). **B)** IgG controls of immunofluorescence stainings of murine fractalkine (green), Ly6G (red) and DNA marker DAPI (blue) in WT lung tissue, 24 hours after LPS (original magnification 20x; one representative image is demonstrated; n=3). Representative immunofluorescence images of IgG controls from human pRIPK1, pRIPK3 respectively pMLKL (all green), CD66b (red) and cell DNA marker DAPI (blue) are shown (original magnification 40x; one representative image is demonstrated; n=3).

Supplemental Figure 4

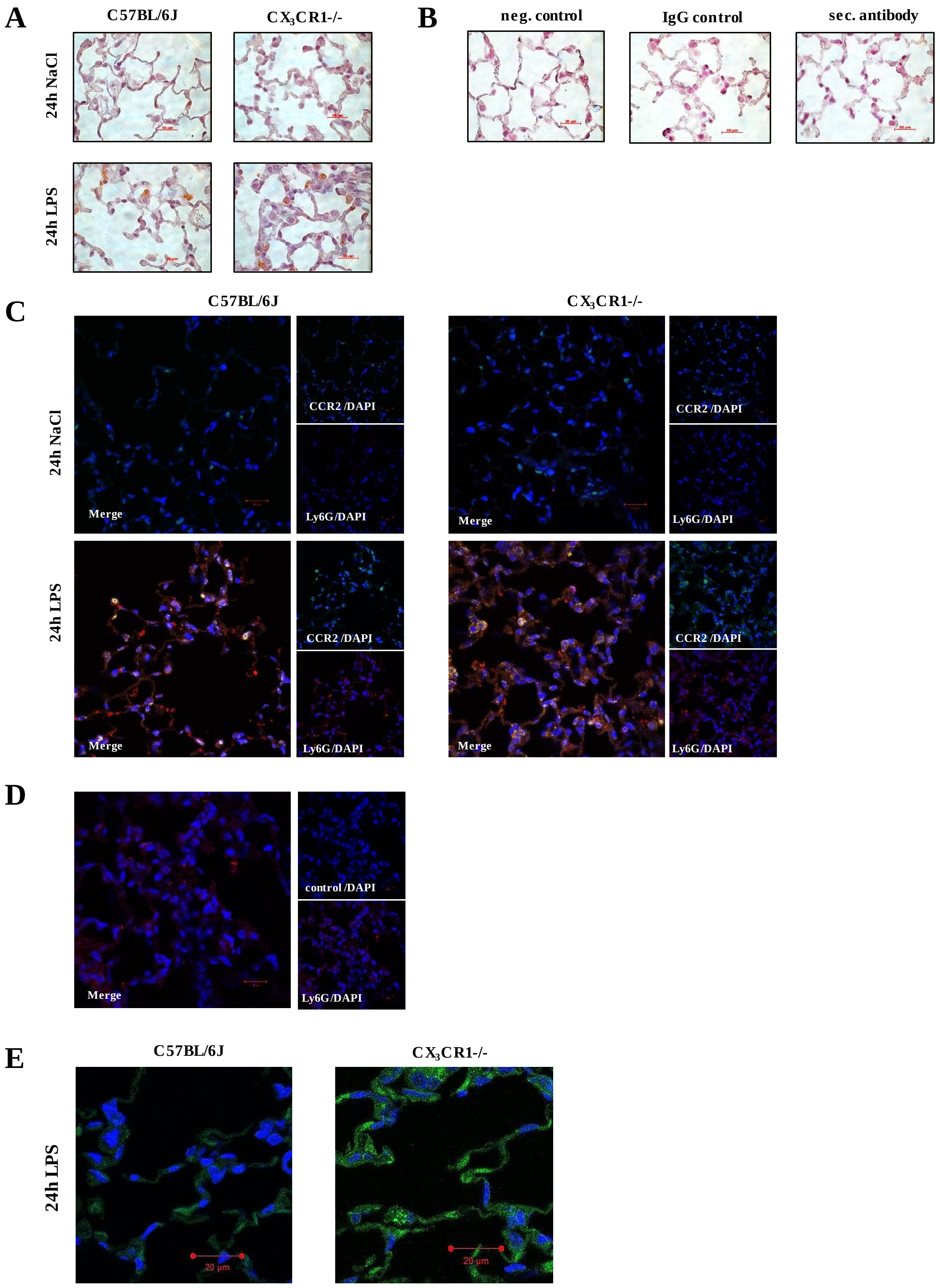

Supplemental figure 4. A) PMN infiltration into the lungs of C57BL/6J and CX_3CR1-/- mice was also quantified by immunohistochemistry. PMNs were stained with a specific marker (rat anti-mouse neutrophil; clone RB6-8C5) and appeared brown (original magnification 100x; one representative image of four is shown; n=4). **B)** Negative controls, IgG- and secondary antibody staining served as controls (original magnification 100x; one representative image of four is shown; n=4). **C)** CCR2-positive PMNs were evaluated by immunofluorescence staining. CCR2 were tackled by a specific antibody and appear (green) and Ly6G-positive leukocytes appeared red (original magnification 63x; one representative image of four is shown; n=4). **D)** Unspecific IgG antibody served as control staining for the CCR2 immunofluorescence experiments (original magnification 63x; one representative image of four is shown; n=4). E) Fluorescein TUNEL staining in the pulmonary tissue of C57BL/6J and CX_3CR1-/- mice 24 hours after LPS exposure. Dead cells were stained with fluorescein green (original magnification 100x, one representative image is shown; n=4).
